# Effects of Selenium Supplementation on Graves' Disease: A Systematic Review and Meta-Analysis

**DOI:** 10.1155/2018/3763565

**Published:** 2018-09-26

**Authors:** Huijuan Zheng, Junping Wei, Liansheng Wang, Qiuhong Wang, Jing Zhao, Shuya Chen, Fan Wei

**Affiliations:** Department of Endocrinology, Guang'anmen Hospital, China Academy of Chinese Medical Sciences, No. 5 Beixiange Street, Xicheng District, Beijing, 100053, China

## Abstract

Low selenium status is associated with increased risk of Graves' disease (GD). While several trials have discussed the efficacy of selenium supplementation for thyroid function, in GD patients, the effectiveness of selenium intake as adjuvant therapy remains unclear. In this systematic review and meta-analysis, we aimed to determine the efficacy of selenium supplementation on thyroid function in GD patients. Two reviewers searched PubMed, Web of Science, the Cochrane Central Register of Controlled Trials, and four Chinese databases for studies published up to October 31, 2017. RCTs comparing the effect of selenium supplementation on thyroid hyperfunction in GD patients on antithyroid medication to placebo were included. Serum free thyroxine (FT4), free triiodothyronine (FT3), thyrotrophic hormone receptor antibody (TRAb), and thyroid-stimulating hormone (TSH) levels were assessed. Ten trials involving 796 patients were included. Random-effects meta-analyses in weighted mean difference (WMD) were performed for 3, 6, and 9 months of supplementation and compared to placebo administration. Selenium supplementation significantly decreased FT4 (WMD=-0.86 [confidence interval (CI)-1.20 to -0.53];* p*=0.756; I^2^=0.0%) and FT3 (WMD=-0.34 [CI-0.66 to -0.02];* p*=0.719; I^2^=0.0%) levels at 3 months, compared to placebo administration; these findings were consistent at 6 but not 9 months. TSH levels were more elevated in the group of patients taking selenium than in the control group at 3 and 6, but not 9 months. TRAb levels decreased at 6 but not 9 months. At 6 months, patients on selenium supplementation were more likely than controls to show improved thyroid function; however, the effect disappeared at 9 months. Whether these effects correlate with clinically relevant measures remains to be demonstrated.

## 1. Introduction

Graves' disease (GD) is an autoimmune thyroid disease which entails the stimulation of the receptor of the thyroid-stimulating hormone (TSH) by the thyrotrophic hormone receptor antibody (TRAb), leading to an increase in thyroid hormone synthesis and release [1]. GD is the main cause of hyperthyroidism in adults and is characterized by subnormal serum TSH levels and increased serum levels of free thyroxine (FT4) and/or triiodothyronine (T3) [2]. In GD, the basal metabolic status of patients is accelerated, resulting in a marked increase in the proportion of free radicals and reactive oxygen species (ROS) [3, 4]. The available literature currently focuses on the role of oxidative stress in the pathogenesis of GD and the protective effects of potent antioxidant systems. This system is accompanied by intracellular antioxidant enzymes, such as superoxide dismutase (SOD), glutathione reductase and glutathione peroxidase (GPx) [5, 6].

The trace element selenium—as an antioxidant—is essential for healthy thyroid hormone metabolism and function [7, 8]. Selenium—in the form of selenocysteine—is incorporated in selenoproteins, such as glutathione peroxidase, which catalyzes the degradation of hydrogen peroxide and lipid hydroperoxide, the production of which is increased in cases of GD [9, 10]. Selenoproteins have an irreplaceable role in thyroid autoimmune processes, and selenium deficiency has a pivotal influence on the initiation and progression of autoimmune thyroid disease [11]. According to a Danish study, the serum selenium levels of patients with newly diagnosed GD were generally lower than those of random controls [12]. In addition, serum selenium levels >120 *μ*g/l were observed in the remission group of one study, indicating a positive correlation with the outcomes of GD [13]. In cases of recurrent hyperthyroidism caused by GD, selenium supplementation can enhance the efficacy of antithyroid drugs and ameliorate thyroid function [14]. Graves' ophthalmopathy (GO) is also associated with low selenium status [15], and relative selenium deficiency should be considered an independent risk factor for GO patients [16]. In a randomized, double-blind, placebo-controlled trial population in Italy, selenium supplementation was found to significantly improve the quality of life and ocular involvement and delay the progression of the disease [17]. Several trials have investigated the effect of selenium intake on thyroid autoantibodies in autoimmune thyroiditis patients. In a 2016 meta-analysis, based on 16 controlled trials, significant lower levels of serum thyroid peroxidase antibody (TPOAb) and thyroglobulin antibody (TgAb) were observed in those with selenium intake [18]. Similar conclusions were reached in another meta-analysis including nine studies, in 2014 [19]. Therefore, an increasing amount of attention has been paid to the role of selenium in the treatment of GD.

In recent times, there have been conflicting reports as to whether selenium supplementation can benefit GD treatment. Some researchers have reported the obvious improvement of selenium supplementation as an adjuvant therapy in patients with GD [14], while others observed only an insignificant modification in the control of hyperthyroidism after selenium intake [20]. To our knowledge, till date, no relevant systemic review or meta-analysis on the efficacy of selenium intake in thyroid function in GD patients has been conducted. Therefore, in this study, we carried out a systemic review and meta-analysis of randomized controlled trials (RCTs), assessing the effect of selenium supplementation on individuals with GD.

## 2. Methods

### 2.1. Data Search and Sources

The following electronic databases were searched to identify relevant studies published from inception to up to October 31, 2017: PubMed, the Cochrane Central Register of Controlled Trials, Web of Science, China National Knowledge Infrastructure, as well as the databases of the Chinese Ministry of Science & Technology (Wangfang), China Biological Medicine, and Chinese Science and Technology Periodicals (VIP). The initial computer-based search terms were conducted using medical subject headings and common text words related to GD, thyrotoxicosis and selenium. The search had no language restriction. In addition, the reference lists of retrieved included trials were searched manually to identify eligible studies. Two review authors (Zheng HJ and Wang LS) independently scanned the titles and abstracts of retrieved records for eligibility. Where discrepancies in opinion existed, they were resolved by consensus or after consulting a third party (Wei JP).

### 2.2. Selection of Studies

The inclusion criteria for this review were RCTs in adults with GD, which compared the efficacy between selenium intake alone or in combination with methimazole (MMI) and placebo intake alone or in combination with MMI, or no treatment. Outcomes included data on preintervention and postintervention thyroid function (e.g., FT3, FT4, TSH, and TRAb levels).

Studies were excluded if they were systematic reviews or meta-analyses, if they were nonrandomized studies, posters, or just abstracts, if they involved participants with a thyroid disease other than GD, or if they reported only categorical data as outcomes.

### 2.3. Data Extraction and Quality Assessment

Full-text articles were evaluated if the inclusion and exclusion criteria could not be found. For studies that fulfilled the inclusion criteria, two reviewers (Wang QH and Zhao J) independently extracted data using standard data extraction templates. Detailed information was extracted: baseline characteristics (the first author, country, and publication date), included population and intervention characteristics (sample size, baseline mean age, sex, and duration of intervention), and outcomes (including at least one of the following levels: FT3, FT4, TSH, and TRAb). Any disagreements were resolved by discussion.

The methodological quality of the included RCTs was evaluated by the Jadad scale in the following domains: randomization, blinding, and the description of withdrawals and dropouts [30]. A cut-off score of 3 was used to indicate high-quality studies, based on previously conducted studies. Data extraction and quality assessment were performed independently by two investigators, and discrepancies in opinions were resolved through discussions and a consensus.

### 2.4. Data Synthesis and Analysis

The effect of treatment in each study was estimated by evaluating changes from the baseline for continuous outcomes. In case that mean ± standard deviations were not provided explicitly, they would be calculated by other available data [31]. For some studies providing more than one time-point outcome during the intervention, data of the last time-point were used for the analyses.

Heterogeneity between the results of different studies was evaluated by the Cochran Q and the I^2^ statistic, with a P value <0.1 and I^2^ >50% indicating statistically significant [32]. A random-effects model would be chosen to pool results if the heterogeneity was significant. Otherwise, a fixed effect model would be used. In case of heterogeneity, subgroup analyses and sensitivity analyses were conducted. Furthermore, Begg's and Egger's tests were used to assess the extent of publication bias. Meta-analysis was conducted using Stata12.0, and the two-sided P value significance level was set at* P* <0.05.

## 3. Results

### 3.1. Characteristics and Quality of the Studies

**Figure 1:**
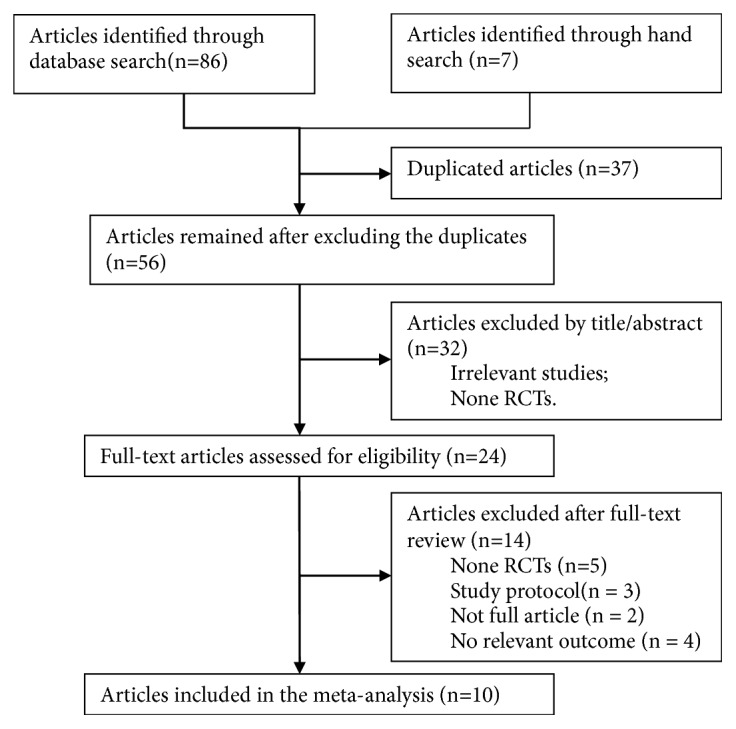
Flow diagram of search and selection processes.

As shown in Figure 1, the initial search strategy identified 86 potentially eligible articles, and seven additional articles were added to the search result by a manual search. Of these 93 records, 37 duplicates of the same articles were excluded and 32 irrelevant studies or none RCTs were discarded. After a detailed assessment of the full text, an additional 14 articles were removed. A total of 10 RCTs met all the eligibility inclusion criteria and were included in the final meta-analysis.

Table 1 summarizes the detailed characteristics and quality of the included RCTs. Most of the included studies were published after 2015 (70.0%, 7/10) and were conducted in China (70.0%, 7/10). The sample sizes of the individual trials ranged from 30 to 241 participants, with a total of 796 participants. The participants' ages ranged from 28 to 45 years at the time of intervention, and the percentage of women ranged from 27% to 90%. All the included trials had two parts: administration of the standard antithyroid drug MMI (doses ranged from 5 mg/d to 30 mg/d) plus selenium (doses ranged from 100 ug/d to 300 ug/d) and MMI with or without placebo. The duration of follow-up ranged from 3 to 9 months. The quality scores of the included RCTs ranged from 2 to 5 points. All the studies adopted the random assignment of patients, and six RCTs did not describe the method of randomization used [21, 22, 24, 25, 28, 29]. Only two of the RCTs were double-blinded [23, 26]. All the RCTs had defined inclusion and exclusion criteria for the patients and provided a statement of the number withdrawals in each group, along with the reasons for the same.

### 3.2. Effects of Selenium Supplementation on FT3 Levels

**Figure 2:**
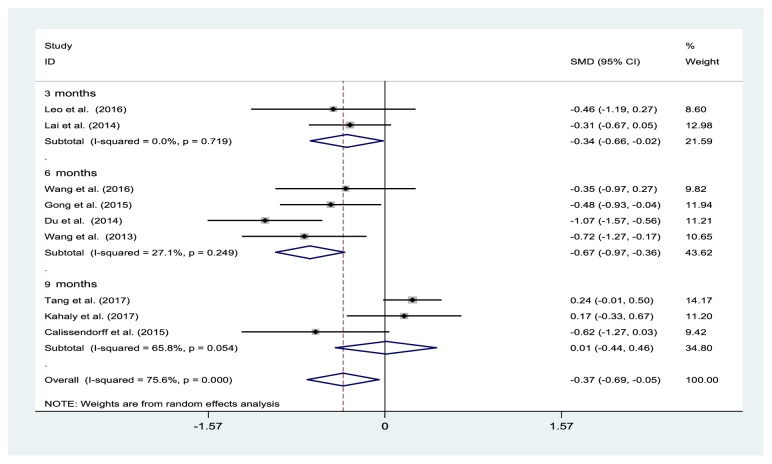
Forest plot of selenium supplementation effects on FT3 levels. CI: confidence interval; SMD: standard mean difference.

The results of nine trials, involving 736 participants, were included in the meta-analysis. Compared with the control group, the pooled estimate showed a significant decrease in the FT3 levels in the selenium treated group, at 3 months (two studies: SMD −0.34%, 95% CI: −0.66% to −0.02%; I^2^=0%, P=0.719) and 6 months (four studies: SMD −0.67%, 95% CI: −0.97% to −0.36%; I^2^=27.1%, P=0.249), but not at 9 months (three studies: SMD 0.01%, 95% CI: −0.44% to −0.46%; I^2^=65.8%, P=0.054) (Figure 2). Sensitivity analyses showed that the pooled results were largely unchanged when each trial was individually removed.

### 3.3. Effects of Selenium Supplementation on FT4 Levels

**Figure 3:**
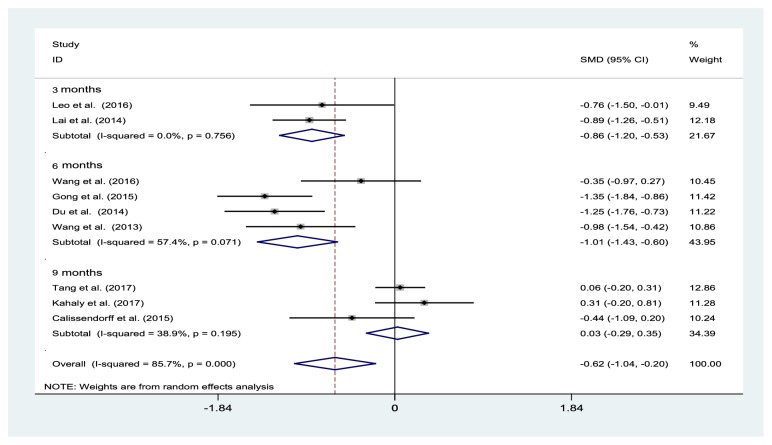
Forest plot of selenium supplementation effects on FT4 levels. CI: confidence interval; SMD: standard mean difference.

The overall pooled results of the studies reporting FT4 levels (nine trials, 736 participants) indicated a significant decrease in the FT4 levels among participants in the selenium treated group when compared to the control participants, at 3 months (two studies: SMD −0.86 mmHg, 95% CI: −1.20 to -0.53 mmHg; P for heterogeneity = 0.756, I^2^ = 0%) and 6 months (four studies: SMD −1.01%, 95% CI: −1.43% to −0.60%; I^2^=57.4%, P=0.071), but not at 9 months (three studies: SMD 0.03%, 95% CI: −0.29% to 0.35%; I^2^=38.9%, P=0.195) (Figure 3). When each trial was individually removed, the overall SMD for the FT4 levels remained largely unchanged.

### 3.4. Effects of Selenium Supplementation on TSH Levels

**Figure 4:**
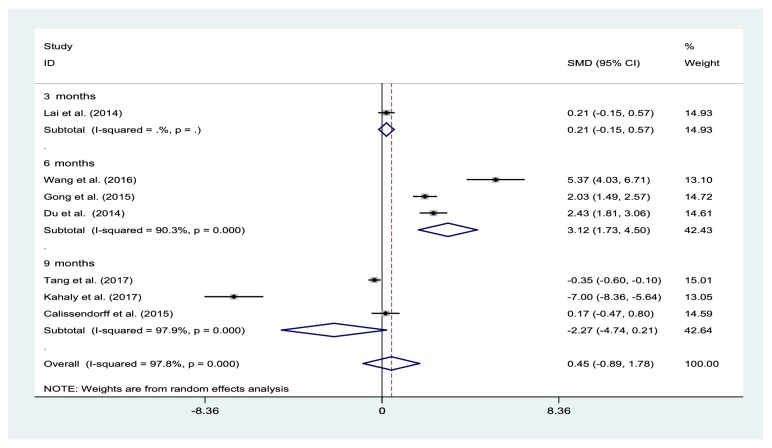
Forest plot of selenium supplementation effects on TSH levels. CI: confidence interval; SMD: standard mean difference.

A total of seven trials, involving 651 participants, showed that selenium supplementation was associated with a significant increase in the TSH levels, at 6 months (three studies: SMD 3.12%, 95% CI: 1.73% to 4.5%; I^2^=90.3%, P=0) when compared to the control group, but not at 3 months (one study: SMD 0.21%, 95% CI: -0.15% to 0.57%) or 9 months (three studies: SMD -2.27%, 95% CI: -4.74% to 0.21%; I^2^=97.8%, P=0) (Figure 4). The overall SMD for the TSH levels remained largely unchanged when each trial was individually removed.

### 3.5. Effects of Selenium Supplementation on TRAb Levels

**Figure 5:**
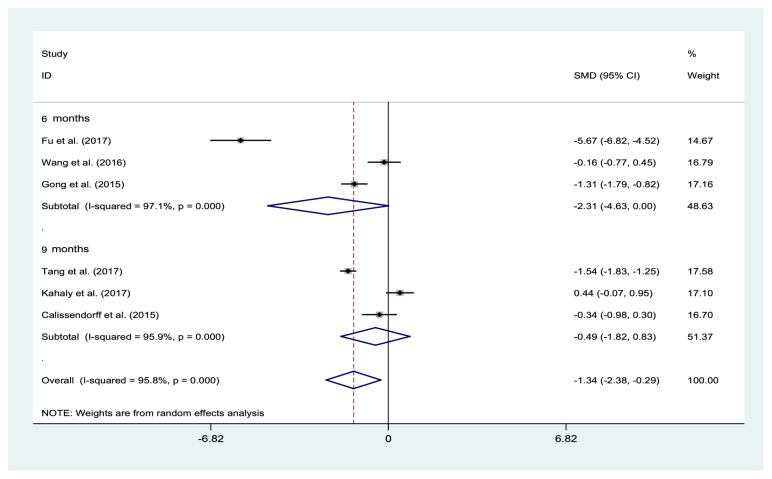
Forest plot of selenium supplementation effects on TRAb levels. CI: confidence interval; SMD: standard mean difference.

Six studies with 736 participants reported the effects of selenium supplementation on TRAb levels. Compared with those in the control group, patients who underwent selenium supplementation displayed a significant decrease in TRAb levels at 6 months (three studies: SMD -2.31%, 95% CI: −4.63% to 0.00%; I2=97.1%, P=0), but not at 9 months (three studies: SMD -1.34%, 95% CI: -2.38% to -0.29%; I2=95.8%, P=0) (Figure 5). The pooled results remained largely unchanged when the trials were individually removed.

### 3.6. Publication Bias

For any of the outcomes, there was no evidence of significant publication bias from either Egger's test or Begg's test (all P >0.05).

## 4. Discussion

GD is associated with an increase in the ROS, diminished endogenous antioxidative capacity, and lowered selenium status. Since selenoenzymes are important for antioxidant defense, adjuvant supplementation with selenium may improve the outcome of patients with GD [5, 6]. However, to date, the clinical trials focusing on selenium supplementation as adjuvant therapy have shown equivocal results. This is the first systematic review and meta-analysis assessing the effectiveness of selenium supplementation when used in addition to standard treatment to normalize the thyroid function in patients with hyperthyroidism caused by GD. This meta-analysis included data from all the eligible RCTs that had a selenium supplementation duration of 3 or 6 months and which produced clinically important and statistically significant effects on the FT4, FT3, TSH, and TRAb levels in patients with GD; nevertheless, selenium supplementation with a duration of 9 months was no more effective in controlling GD than in the control. This may be attributed to the fact that the health effects of selenium have an inextricable U-shaped link with status; only two of the ten trials in this meta-analysis measure sequentially selenium serum levels in GD patients under adjuvant supplemental selenium treatment, so we cannot find out whether selenium supplementation was more effective for those who had lower selenium serum levels by subgroup analysis. However, we may find some supporting evidence from two trials among patients with GD reported selenium status before and following supplementation [20, 23], which showed that the serum levels of selenium at baseline in the range of 109-115ug/l and no selenium deficiency in the vast majority of their patients during and after treatment. As to both of the two studies that failed to show an adjuvant role of selenium in the short-term control of hyperthyroidism, this might be due to selenium supplementation not offering any advantage if selenium intake is adequate, and selenium is likely useful if the patient is selenium-deficient [33].

The positive findings with regards to the FT3, FT4, and TSH levels at 3 and 6 months after selenium supplementation in our meta-analysis are in accordance with the findings of trials examining a wide range of conditions. In one trial which focused on individuals in an area with severe iodine and selenium deficiency, serum T4 and FT3 levels decreased significantly after 2 months of selenium supplementation, but no increase was observed in the serum TSH levels [34]. It has been shown in selenium-deficient rats that protein iodination in the thyroid gland was enhanced, and type I deiodinase activity was inhibited in the absence of selenium, resulting in increased thyroid hormone synthesis and elevated serum T4 and T3 values [35]. Our meta-analysis indicated that selenium supplementation effectively reduces TRAb levels at 6 months. According to the evidence available, a reduction in the circulating TRAb titers is clinically relevant, as TRAb is considered a surrogate marker of hyperthyroidism caused by GD and a risk predictor of relapse at the time of antithyroid drug withdrawal [36]. Epidemiological data suggest an increased prevalence of autoimmune thyroid diseases under conditions of selenium deficiency [12, 37]. Our finding is consistent with those of other meta-analyses supporting selenium supplementation for autoimmune thyroiditis. A systematic review and meta-analysis by Toulis KA et al. [38] reported that selenium supplementation was associated with a significant decrease in the levels of TPOAb in the treatment of Hashimoto's thyroiditis. The immunomodulatory effects of selenium supplementation may be the mechanisms responsible for the treatment of autoimmune thyroid disease [39]. In a mouse model of autoimmune thyroiditis, selenium was found to effectively reduce lymphocytic infiltration of the thyroid and upregulate the regulatory T cells with increased GPx and thioredoxin reductase expressions [40]. Another study showed that selenoprotein K deficiency may decrease Ca(2+) flux during immune cell activation, in mice, causing an impaired immune response [41]. The role of selenium has also been studied in the case of GO. A European multicenter RCT designed by the European Group of Graves' Orbitopathy group evaluated the effect of selenium compared with placebo on patients with GO and found a significant improvement in ophthalmological symptoms and quality of life after a 6-month intervention period [17]. With regards to the beneficial effects of the antioxidant agent—selenium—in GO, a possible cellular mechanism through which selenium acts was recently proposed, which states that it may inhibit cell proliferation and the secretion of proinflammatory cytokines such as tumor necrosis factor alpha and interferon gamma [42].

Although some aspects of this meta-analysis could be improved, it also has several strengths. It is, to date, the first comprehensive analysis to quantitatively assess the impact of selenium supplementation on hyperthyroidism caused by GD. In addition, relevant trials were identified using a scientific and comprehensive search strategy. Finally, this meta-analysis helps to increase the available evidence on selenium for use in the endocrine field. Data from a 2016 Italian questionnaire study [43] showed that 85.2% of endocrinologists considered using selenium in daily clinical practice for thyroid disease and about 21.5% of respondents would recommend selenium for GD without GO and 25% for GD with mild GO. Therefore, selenium supplementation is prescribed, despite not being recommended in international guidelines for the management of hyperthyroidism.

Our study has some limitations. First, the available evidence on the routine use of selenium supplementation in the treatment of GD patients is insufficient and the corresponding RCTs were limited by their small sample sizes. Second, substantial heterogeneity was detected in the pooled results of TSH and TRAb among the included trials. As some of the studies did not provide enough clinical information, we could not perform thorough analyses to explore the source of heterogeneity. Most of the studies included did not discuss the disease-process and did not report selenium status of the study populations, using not exactly the same selenocompounds and dosages. With the aggravation of the disease, the absorption rate and the effect of selenium would reduce. Meanwhile, the absorption in different selenocompounds varied and the number of studies is not yet sufficiently high to be able to compare the effects of different selenocompounds and dosages from these divergent results. So it may lead to high heterogeneity. Nonetheless, a random-effects model was adopted to pool estimations when appropriate, so as to provide the most conservative estimates. Furthermore, sensitivity analyses revealed that the results would not have changed when the trials were individually removed. Third, despite most of studies being adjudged as having a low risk of selection bias and reporting bias, inadequate reporting of the proper allocation concealment and blinding method may increase the risk of selection bias and attribution bias. Therefore, the results should be interpreted with caution.

Future research on selenium supplementation in GD patients should focus on RCTs with different time-points after selenium intake. In addition, these trials should focus on the selenium status of the study populations. Finally, some of the trials included in our meta-analysis had a moderate or high risk of bias, mainly due to the small sample sizes and unclear allocation concealment and blinding method. To confirm the positive outcomes observed in our review, methodologically rigorous trials with large samples are necessary.

Compared with our previous meta-analysis of the effect of telephone call intervention on glycemic control and other cardiovascular risk factors in type 2 diabetes mellitus patients [44], this meta-analysis assessed the effect of selenium supplementation on individuals with GD. Therefore, the purposes, interventions, patients, and outcomes of the two meta-analysis are different.

## 5. Conclusions

Our meta-analysis shows that adjuvant selenium supplementation can enhance the restoration of biochemical euthyroidism and might benefit patients with GD. The positive findings should be reproduced in larger methodological trials before selenium can be included in standard clinical treatment, in international guidelines.

## Figures and Tables

**Table 1:** Characteristics and quality of the included randomized controlled trials.

***Study***	***Country***	***N (Case:***	***Intervention***		***Age***	%	***Duration***	***Main***	***Jadad***
***(reference number)***		***Control)***	***Case***	***Control***	***(years)***	***female***	***in months***	***Outcome*** ***measures***	***scores***
Tang et al. [21], 2017	China	241(121/120)	MMI 20 mg/d + Selenious yeast tablet 200 ug/d	MMI 20 mg/d	28	75	9	FT3, FT4, TSH, TRAb	2

Fu et al. [22], 2017	China	60 (30/30)	MMI 15-30 mg/d + Selenious yeast tablet 300 ug/d	MMI 15-30 mg/d	38	58	6	TRAb	2

Kahaly et al. [23], 2017	Germany	61 (29/32)	MMI 10 mg/d + Sodium selenite 300 ug/d	MMI 10 mg/d +placebo	45	27	9	FT3, FT4, TSH, TRAb	5

Leo et al. [20], 2016	Italy	30 (15/15)	MMI 5-30 mg/d + L-selenomethionine 166 ug/d	MMI 5-30 mg/d	40	90	3	FT3, FT4,	3

Wang et al. [24], 2016	China	41 (21/20)	MMI 18 mg/d + Sodium selenite 200 ug/d	MMI 18 mg/d	38	82	6	FT3, FT4, TSH, TRAb	2

Gong et al. [25], 2015	China	80 (40/40)	MMI 15-30 mg/d + Selenious yeast tablet 200 ug/d	MMI 15-30 mg/d	36	56	6	FT3, FT4, TSH, TRAb	2

Calissendorff et al. [26], 2015	Sweden	38 (19/19)	MMI 30 mg/d + Selenious yeast tablet 200 ug/d	MMI 30 mg/d +placebo	39	82	9	FT3, FT4, TSH, TRAb	5

Lai et al. [27], 2014	China	120 (60/60)	MMI 5-30 mg/d + Selenious yeast tablet 100 ug/d	MMI 5-30 mg/d	40	77	3	FT3, FT4, TSH, TRAb	3

Du et al. [28], 2014	China	70 (38/32)	MMI 20 mg/d + Selenious yeast tablet 150 ug/d	MMI 20 mg/d	36	59	6	FT3, FT4, TSH	2

Wang et al. [29], 2013	China	55 (30/25)	MMI 20 mg/d + Selenious yeast tablet 200 ug/d	MMI 20 mg/d	37	27	6	FT3, FT4	2

MMI: methylimidazole; FT4: free thyroxine; FT3: free triiodothyronine; TRAb: thyrotrophic hormone receptor antibody; TSH: thyroid-stimulating hormone.

## List of Abbreviations

GD: Graves' disease
TSH: Thyroid-stimulating hormone
FT4: Free thyroxine
FT3: Free triiodothyronine
ROS: Reactive oxygen species
GPx: Glutathione peroxidase
SOD: Superoxide dismutase
GO: Graves' ophthalmopathy
TPOAb: Thyroid peroxidase antibody
TgAb: Thyroglobulin antibody
RCT: Randomized control trial
MMI: Methimazole
TRAb: Thyrotrophic hormone receptor antibody.

## References

[1] Ross D. S., Burch H. B., Cooper D. S. (2016). 2016 American Thyroid Association Guidelines for Diagnosis and Management of Hyperthyroidism and Other Causes of Thyrotoxicosis. *Thyroid*.

[2] Burch H. B., Cooper D. S. (2015). Management of Graves Disease: A Review. *Journal of the American Medical Association*.

[3] Komosinska-Vassev K., Olczyk K., Kucharz E. J., Marcisz C., Winsz-Szczotka K., Kotulska A. (2000). Free radical activity and antioxidant defense mechanisms in patients with hyperthyroidism due to Graves' disease during therapy. *Clinica Chimica Acta*.

[4] Bednarek J., Wysocki H., Sowinski J. (2004). Oxidation products and antioxidant markers in plasma of patients with Graves' disease and toxic multinodular goiter: Effect of methimazole treatment. *Free Radical Research*.

[5] Marcocci C., Leo M., Altea M. A. (2012). Oxidative stress in graves' disease. *European Thyroid Journal*.

[6] Žarković M. (2012). The role of oxidative stress on the pathogenesis of Graves' disease. *Journal of Thyroid Research*.

[7] Köhrle J. (2005). Selenium and the control of thyroid hormone metabolism. *Thyroid*.

[8] Rayman M. P. (2000). The importance of selenium to human health. *The Lancet*.

[9] Duntas L. H. (2012). The evolving role of selenium in the treatment of Graves' disease and ophthalmopathy. *Journal of Thyroid Research*.

[10] Schomburg L. (2012). Selenium, selenoproteins and the thyroid gland: Interactions in health and disease. *Nature Reviews Endocrinology*.

[11] Ventura M., Melo M., Carrilho F. (2017). Selenium and thyroid disease: From pathophysiology to treatment. *International Journal of Endocrinology*.

[12] Bülow Pedersen I., Knudsen N., Carlé A. (2013). Serum selenium is low in newly diagnosed Graves' disease: A population-based study. *Clinical Endocrinology*.

[13] Wertenbruch T., Willenberg H. S., Sagert C. (2007). Serum selenium levels in patients with remission and relapse of Graves' disease. *Medicinal Chemistry*.

[14] Wang L., Wang B., Chen S. (2016). Effect of selenium supplementation on recurrent hyperthyroidism caused by graves’ disease: a prospective pilot study. *Hormone and Metabolic Research*.

[15] Dehina N., Hofmann P. J., Behrends T., Eckstein A., Schomburg L. (2016). Lack of association between selenium status and disease severity and activity in patients with graves' ophthalmopathy. *European Thyroid Journal*.

[16] Khong J. J., Goldstein R. F., Sanders K. M. (2014). Serum selenium status in Graves' disease with and without orbitopathy: A case-control study. *Clinical Endocrinology*.

[17] Weissel M. (2011). Selenium and the course of mild Graves' orbitopathy. *New England Journal of Medicine*.

[18] Wichman J., Winther K. H., Bonnema S. J., Hegedüs L. (2016). Selenium Supplementation Significantly Reduces Thyroid Autoantibody Levels in Patients with Chronic Autoimmune Thyroiditis: A Systematic Review and Meta-Analysis. *Thyroid*.

[19] Fan Y., Xu S., Zhang H. (2014). Selenium supplementation for autoimmune thyroiditis: a systematic review and meta-analysis. *International Journal of Endocrinology*.

[20] Leo M., Bartalena L., Rotondo Dottore G. (2017). Effects of selenium on short-term control of hyperthyroidism due to Graves’ disease treated with methimazole: results of a randomized clinical trial. *Journal of Endocrinological Investigation*.

[21] Hui T., Hui S., Zhen S. (2017). Observation of TPO-Ab and TRAb in blood of the patients with graves disease after iodine 131 treatment and selenium yeast. *Journal of Medical Theory & Practice*.

[22] Huan F., Liyun W. (2017). The clinical research of selenium yeast joint methimazole in the treatment for graves disease with hyperthyroidism. *Chinese and Foreign Medical Research*.

[23] Kahaly G. J., Riedl M., König J., Diana T., Schomburg L. (2017). Double-blind, placebo-controlled, randomized trial of selenium in graves hyperthyroidism. *The Journal of Clinical Endocrinology & Metabolism*.

[24] Wang L., Wang B., Chen S. (2016). Effect of selenium supplementation on recurrent hyperthyroidism caused by graves’ disease: a prospective pilot study. *Hormone and Metabolic Research*.

[25] Gong M., Wang A. (2015). Clinical study on effct of selenium combining with methimazole in graves’ disease patients with hyperthyroidism. *Chinese Journal of Traditional Medical Science and Technology*.

[26] Calissendorff J., Mikulski E., Larsen E. H., Möller M. (2015). A prospective investigation of graves’ disease and selenium: thyroid hormones, auto-antibodies and self-rated symptoms. *European Thyroid Journal*.

[27] Lai J. (2014). Efficacy of selenious yeast tablets combined with methimidazole in the treatment of graves’ disease. *Chinese Journal of Pharmacoepidemiology*.

[28] Ying-Hong U. D., Mao R. R., Endocrinology D. O. (2013). Clinical research on the selenium combination with methimazole in treatment of Graves’ disease. *Journal of Hunan University of Chinese Medicine*.

[29] Wang J., Zhang W., Li J. (2013). The effect of selenium on the level of serum TPOAb and TGAb in patients with Graves’ disease. *Chinese Journal of Integrative Medicine on Cardio-/cerebrovascular Disease*.

[30] Jadad A. R., Moore R. A., Carroll D. (1996). Assessing the quality of reports of randomized clinical trials: Is blinding necessary?. *Controlled Clinical Trials*.

[31] Wiebe N., Vandermeer B., Platt R. W., Klassen T. P., Moher D., Barrowman N. J. (2006). A systematic review identifies a lack of standardization in methods for handling missing variance data. *Journal of Clinical Epidemiology*.

[32] Higgins J. P. T., Thompson S. G. (2002). Quantifying heterogeneity in a meta-analysis. *Statistics in Medicine*.

[33] Rayman M. P. (2012). Selenium and human health. *The Lancet*.

[34] Contempré B., Duale N. L., Dumont J. E., Ngo B., Diplock A. T., Vanderpas J. (1992). Effect of selenium supplementation on thyroid hormone metabolism in an iodine and selenium deficient population. *Clinical Endocrinology*.

[35] Bates J. M., Spate V. L., Steven Morris J., St Germain D. L., Galton V. A. (2000). Effects of selenium deficiency on tissue selenium content, deiodinase activity, and thyroid hormone economy in the rat during development. *Endocrinology*.

[36] Bartalena L. (2013). Diagnosis and management of Graves disease: a global overview. *Nature Reviews Endocrinology*.

[37] Wu Q., Rayman M. P., Lv H. (2015). Low population selenium status is associated with increased prevalence of thyroid disease. *Journal of Clinical Endocrinology & Metabolism*.

[38] Toulis K. A., Anastasilakis A. D., Tzellos T. G., Goulis D. G., Kouvelas D. (2010). Selenium supplementation in the treatment of Hashimoto's thyroiditis: a systematic review and a meta-analysis. *Thyroid Official Journal of the American Thyroid Association*.

[39] Duntas L. H. (2010). Selenium and the thyroid: A close-knit connection. *The Journal of Clinical Endocrinology & Metabolism*.

[40] Wang W., Xue H., Li Y. (2015). Effects of selenium supplementation on spontaneous autoimmune thyroiditis in NOD.H-2h4 mice. *Thyroid*.

[41] Verma S., Hoffmann F. W., Kumar M. (2011). Selenoprotein K knockout mice exhibit deficient calcium flux in immune cells and impaired immune responses. *The Journal of Immunology*.

[42] Rotondo Dottore G., Leo M., Casini G. (2017). Antioxidant Actions of Selenium in Orbital Fibroblasts: A Basis for the Effects of Selenium in Graves' Orbitopathy. *Thyroid Official Journal of the American Thyroid Association*.

[43] Negro R., Attanasio R., Grimaldi F., Marcocci C., Guglielmi R., Papini E. (2016). A 2016 italian survey about the clinical use of selenium in thyroid disease. *European Thyroid Journal*.

[44] Wei J., Zheng H., Wang L. (2017). Effects of telephone call intervention on cardiovascular risk factors in type 2 diabetes mellitus: A meta-analysis. *Journal of Telemedicine Telecare*.

